# Fabrication of Microfluidic Devices for Emulsion Formation by Microstereolithography

**DOI:** 10.3390/molecules26092817

**Published:** 2021-05-10

**Authors:** Max J. Männel, Elif Baysak, Julian Thiele

**Affiliations:** Leibniz-Institut für Polymerforschung Dresden e.V., Hohe Str. 6, 01069 Dresden, Germany; max5191max@googlemail.com (M.J.M.); baysak@ipfdd.de (E.B.)

**Keywords:** projection micro-stereolithography, microfluidics, droplets, emulsions, three-dimensional, 3D printing, additive manufacturing

## Abstract

Droplet microfluidics—the art and science of forming droplets—has been revolutionary for high-throughput screening, directed evolution, single-cell sequencing, and material design. However, traditional fabrication techniques for microfluidic devices suffer from several disadvantages, including multistep processing, expensive facilities, and limited three-dimensional (3D) design flexibility. High-resolution additive manufacturing—and in particular, projection micro-stereolithography (PµSL)—provides a promising path for overcoming these drawbacks. Similar to polydimethylsiloxane-based microfluidics 20 years ago, 3D printing methods, such as PµSL, have provided a path toward a new era of microfluidic device design. PµSL greatly simplifies the device fabrication process, especially the access to truly 3D geometries, is cost-effective, and it enables multimaterial processing. In this review, we discuss both the basics and recent innovations in PµSL; the material basis with emphasis on custom-made photopolymer formulations; multimaterial 3D printing; and, 3D-printed microfluidic devices for emulsion formation as our focus application. Our goal is to support researchers in setting up their own PµSL system to fabricate tailor-made microfluidics.

## 1. Introduction

Microfluidics is a versatile tool for analytical chemistry, system integration, and material design, with droplet microfluidics being one of the most prominent examples. Microfluidics focuses on the manipulation and generation of monodisperse droplets inside microchannels [1]. Microfluidic droplet formation has been used extensively for manufacturing advanced materials [2], in drug delivery [3], and in food applications [4]. Conventionally, microfluidic devices are fabricated using a combination of photo- and soft lithography [5], glass-capillary assembly [6], hot embossing [7], and injection molding [8,9]. However, these techniques share several drawbacks. They are time-consuming and require experienced users and a nearly dust-free environment for their successful fabrication. Moreover, to achieve complex three-dimensional (3D) microchannel structures, multistep processes are commonly required to build up 3D microchannels layer-by-layer. Finally, in microchannel systems that only extend in a single plane, the microchannel surface needs to be tailored for individual applications, e.g., emulsion formation with tailored compartmentalization.

Likewise, the materials conventionally used in microfluidic device fabrication suffer several disadvantages. Polydimethylsiloxane (PDMS) swells in many organic solvents, whereas poly(methyl methacrylate) (PMMA) exhibits both poor chemical resistance and poor gas permeability, which is a key requirement for culturing cells [10]. Cyclic olefin copolymers may lack sufficient bonding [11], and parylene can suffer from poor adhesion in wet environments [12]. These shortcomings of both fabrication methods and material properties have hampered the critical step of transitioning microfluidics into industrial applications. Thus, a sustained need exists for a fabrication method that overcomes these drawbacks and offers reliable microfabrication covering a broad range of material properties and microchannel architectures.

In recent years, additive manufacturing (AM), which is also referred to as 3D printing, has attracted intensive attention as a new fabrication method for microfluidic devices. It offers several advantages over the aforementioned conventional fabrication methods. A large library of materials is available for AM, and support materials can be easily removed (e.g., by cutting or dissolution in solvents). In addition, AM requires no specialized cleanroom facilities that provide a dust-free environment, and state-of-the-art commercial 3D printers have small footprints. Furthermore, the digitalization of flow cell designs has opened new opportunities for the simple distribution of data and designs, which makes them accessible to a wide range of consumers worldwide. AM has the potential to become the dominant flow-cell fabrication method in microfluidics because of these advantages. Several excellent reviews have already highlighted the potential of AM in microfluidic device fabrication [13,14,15]. In the present review, we specifically focus on one high-resolution AM technology—projection micro-stereolithography (PµSL)—and its application in droplet microfluidics. With further advances in material design, 3D-printer software, and 3D-printer hardware, we believe that PµSL will supplant conventional flow-cell fabrication methods within the next few years.

## 2. Micro-Stereolithography

**Scheme 1:**
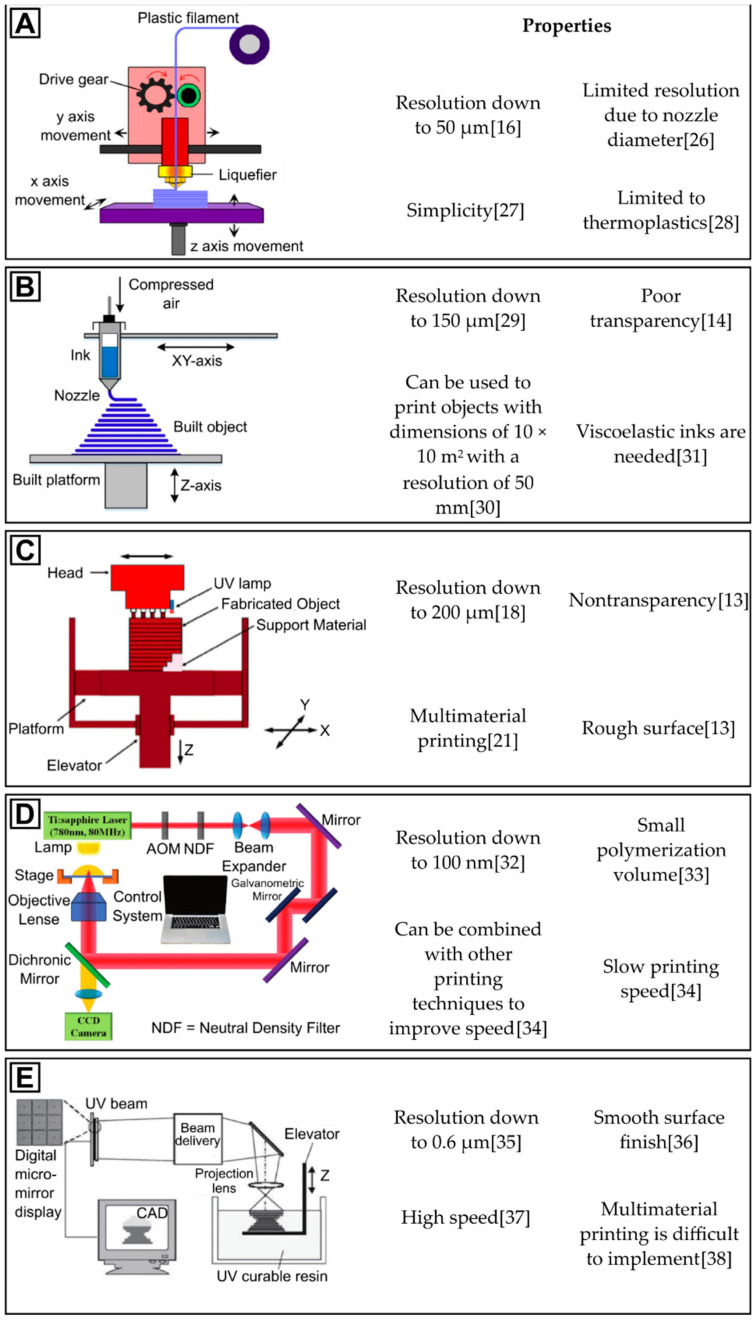
Refs. [26,27,28,29,30,31,32,33,34,35,36,37,38] 3D-printing techniques and their characteristics properties: (**A**) Fused deposition modeling (adapted with permission from Akil et al. [39]), (**B**) direct ink writing (adapted with permission from Peretyagin et al. [40]), (**C**) multijet modeling (adapted with permission from Xu et al. [41]), (**D**) two-photon polymerization (adapted with permission from Xu et al. [42]), and (**E**) projection micro-stereolithography (adapted with permission from Zhang et al. [35]).

Several 3D printing technologies have been used for microfluidic device fabrication, including extrusion-based fused deposition modeling (FDM) [16] and direct ink writing (DIW) [17], multijet modeling (MJM) [18], two-photon polymerization (2PP) [19], and PµSL [20], each with a distinct set of advantages and drawbacks (Scheme 1). For instance, although extrusion-based printing is widely used because of its experimental simplicity, a key requirement is that the material be a thermoplastic. FDM is not suitable for high-throughput fabrication using a single print head because it involves the placement of fluidized material at one individual position at a time. By contrast, in PµSL, the whole layer is illuminated and polymerized simultaneously. However, the speed of FDM can be improved through the use of several print heads that are assembled into an array. In addition, the diameter of the extrusion nozzle largely controls the minimal process resolution, which cannot be simply exchanged. MJM printing is based on a photocurable polymer, has a high resolution with a reported channel width of 200 µm [18], and multimaterial printing can be easily implemented [21]. However, the relatively high cost of the associated printers may hinder the broader application of MJM printing. 2PP provides the same resolution that is achieved with conventional photolithography, e.g., for microfluidic master device fabrication. It requires a laser system in which the energy of two photons is combined to initiate the polymerization of a resin. Because of the nature of this process, the photopolymerization is confined to the focal plane of the laser, and the photopolymerization is induced by moving the focal point of the beam [22]. A Ti:sapphire system with a typical repetition rate between 1 kHz and 80 MHz is the most common laser source [23]. In this process, 2PP addresses one voxel after another; the high frequency of the laser beam enables this process to print a structure in a nearly continuous fashion. However, 2PP is likewise time-consuming and it requires transparent liquids, which largely precludes the use of materials that are loaded with light-scattering additives, such as ceramic nanoparticles. In addition, 2PP systems are not only high-end, but also high-cost, which is not affordable for many research groups. We recommend two excellent reviews for readers who are interested in further details of 2PP [24,25].

PµSL has garnered intensive attention in the field of microfluidic device fabrication, because it is capable of providing high resolution with a small minimal feature size in the range from 20 µm to several hundred micrometers, is compatible with a diverse material library (e.g., for achieving tailored transparency), and is rapid as well as inexpensive (commercial PµSL printers are available for less than 10,000 USD). Section 3.2. provides a detailed description of parameter control in PµSL. The laser scanner in a stereolithography (SL) system is replaced with either a liquid crystal display (LCD) [43], a digital micromirror device (DMD) [44], or liquid crystals on silicon (LcoS) [45]. The advantage of PµSL is that any design is printed layer-by-layer instead of individually addressing one voxel at a time, which enables a higher printing speed than can be achieved with standard SL [35,46]. We recommend the recent review by Ge et al. for readers that have more interest in the resolution of the different printing techniques [47].

Before a discussion the details of microfluidic device design for droplet formation via PµSL, in the following section we highlight recent advances in PµSL, provide an overview of custom-made 3D printers based on PµSL as well as “homemade” photopolymer formulations, and highlight developments in multimaterial printing using PµSL.

### 2.1. Recent Innovations in Additive Manufacturing of Microscale Polymer Structures

**Figure 1:**
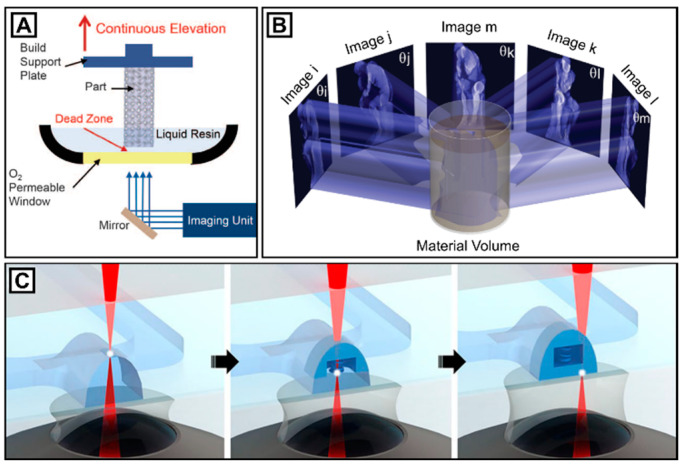
Recent innovations in additive manufacturing based on resin vats. (**A**) Schematic of continuous liquid interface production (CLIP) enabling continuous photopolymer processing at higher speed than conventional PµSL due to an oxygen inhibition layer at the bottom of the resin vat. Adapted with permission from Tumbleston et al. [37] (**B**) Computed axial lithography (CAL) system based on computed 3D exposure of a photoresponsive material from different angles. Adapted with permission from Kelly et al. [53] (**C**) Illustration of in situ direct laser writing (*is*DLW). Focused femtosecond laser pulses, the spatially controlled photopolymerization, and the cured photomaterial are represented as red, white, and blue, respectively. Adapted with permission from Lamont et al. [54].

Sochol et al. highlighted SL as promising technology for fabricating microfluidic devices capable of manufacturing sub-100 µm microchannels [48]. Different approaches have also been proposed to further reduce the processing time in SL printing from hours to even minutes. Figure 1 shows the processes of continuous liquid interface production (CLIP), 2PP, and computed axial lithography (CAL), which are currently the most prominent examples in this field. Newly introduced xolography technology is also promising for high-resolution printing with reduced production times (Figure 1).

The CLIP technique is based on a conventional PµSL setup, but with an oxygen-permeable window at the bottom of the resin vat, which inhibits free-radical photopolymerization due to atmospheric oxygen. The oxygen forms an uncurable layer (dead zone) between the oxygen-permeable window and the resin. This dead zone is reloaded with fresh photopolymer by the movement of the build platform, thereby obviating the need for an iterative layer-by-layer process. CLIP also enables continuous 3D production with a tailored surface finish. For instance, DeSimone and coworkers printed layer-less 3D gyroid and argyle structures at a print speed of 500 mm h^−1^, achieving a height of approx. 5 cm in less than 10 min [37]. Huang et al. introduced supramolecular, biocompatible alginate/polyacrylamide shape-memory hydrogels for biomedical and tissue engineering via CLIP technology [49]. In addition, 3D-printed bioresorbable vascular scaffolds [50] and microneedles for transdermal drug delivery of therapeutics have also been recently introduced [51]. He et al. studied different machine learning techniques for modeling and predicting the proper printing speed in the CLIP process and validated their findings with experimental data [52].

2PP, which is also known as direct laser writing (DLW), can be classified into two subcategories: in situ DLW (*is*DLW) [54,55,56] and dip-in DLW [57]. In the *is*DLW process, conventionally manufactured microfluidic channels can be filled with a photocurable liquid phase, followed by DLW printing inside the microchannel. In the dip-in DLW process, a liquid photoresist itself is used as the immersion liquid between a microscope lens and a substrate, which enables both millimeter-scale overall heights and submicrometer feature sizes. As an example of application, Giacomo et al. introduced deployable microtraps that were similar to miniaturized lobster pots to separate bacteria in a liquid suspension [58]. Dip-in DLW has also been used for designing biocages for drug delivery [59], microfluidic filtration systems for cell sorting [60], and for fabricating 3D structures comprising multiple materials [61]. 2PP is a comparatively young technology in AM. It relies on using focused femtosecond laser pulses to initiate the controlled polymerization of photocurable resins through two-photon absorption to directly write the desired pattern with an exceptionally high (sub-100 nm) resolution [56,62,63]. However, 2PP has drawbacks that are related to its limited printing volume and lengthy processing time. Thus, rather than manufacturing a total device via 2PP, researchers often combine this technology with other material processing techniques, e.g., to fabricate parts of flow cells at high resolution [15,64]. Recently, the group of Chen developed a femtosecond laser projection technique, which improved the process time by parallelized printing [34].

The third example is CAL, which was inspired by computed tomography, in which 2D image projections are constructed through a material from different angles. Using this concept to illuminate a photopolymer formulation enables polymer materials to be manufactured in a volumetric fashion [53]. The photocurable material prefilled into the illumination volume remains static during the photopolymerization process because of the absence of any moving part in the printing process; therefore, highly viscous resins can be processed. As another feature of CAL, prefabricated objects can be used as substrates in the printing volume, enabling step-by-step multimaterial fabrication via AM. Moreover, CAL is several orders of magnitude faster than other techniques; a centimeter-scale geometry can be printed in less than 1 min [53,65]. However, this fairly new technique is currently unable to 3D-print hollow structures at high resolution, which would render it suitable for printing microfluidics.

Recently, Hecht et al. introduced xolography [55]. Its theoretical approach is similar to that of CAL and it relies on volumetric 3D printing [34,66,67]. The authors used a dual-color illumination technique to induce local polymerization by intersecting light beams of different wavelengths in combination with photoswitch molecules. They reported that their approach has a resolution that is ten times greater than that of CAL, and it is five orders of magnitude faster than 2PP. On the basis of their initial report [55], this technique is promising for combining both high resolution and fast processing, but has not yet been evaluated for use in the field of microfluidics.

### 2.2. Custom-Made PµSL 3D Printers and Photopolymer Formulations

While commercially available PµSL printers and resins have greatly simplified the first implementation of AM in microfluidic device fabrication, the current trend favors custom-made solutions on both the process side and material side. The advantages of custom-made printers and resins over commercial ones are obvious. For instance, not all commercial PµSL printers can process homemade resins because of built-in restrictions, e.g., to promote the distribution of photopolymer formulations from the printer’s manufacturer. Whereas an all-in-one solution of a resin library and a PµSL printer by a vendor may be beneficial for new users in the field of PµSL because of the expected perfect adaption of resin properties to the printing process, explorative research will likely require open-source solutions. Moreover, the ability to control the material properties of 3D-printed objects is rather limited because the exact composition of most commercial resins for PµSL is unknown. For instance, many commercial resins only provide limited transparency because of nanoparticles inside the photopolymer formulation, whereas transparency is mandatory when producing microfluidic devices that enable flow-inspection experiments using UV–Vis-based optical techniques.

These disadvantages have motivated researchers to develop custom-made solutions at both the 3D-printer and resin level to gain full control over essential printing and material parameters, such as the process speed, multimaterial-processing capability, minimal feature size, transparency, elasticity, and biocompatibility. In 2012, Zheng et al. proposed a custom-built PµSL printer with a resolution of 1.3 µm per pixel at the focal plane [45]. Their system was based on LcoS, and the exposure energy of the LED source ranged from 1 mW cm^−2^ to 100 mW cm^−2^. With this setup, complex 3D structures, such as tetradecahedrons, were successfully manufactured. Gong et al. reported a custom-made µSL printer based on a DMD that provided a lateral accuracy of 7.6 µm and used a resin formulation to fabricate flow channels with a cross-section of 18 μm × 20 µm [68]. In 2019, Najafi and coworkers [69] introduced a PµSL process with a lateral resolution of not more than 5 µm and a reduced overall printer size as compared with that of Gong et al. [68]. They then used the customized PµSL printer to manufacture helical and hollow structures as a proof-of-principle to provide micron-scale polymer objects with complex geometry [69]. A common feature of the aforementioned custom-made solutions is that they provide open process parameters and are not restricted in their choice of material, thus enabling the user to tailor physicochemical and mechanical material properties for a target application.

In general, the typical material basis for any of the aforementioned examples in PµSL is a photopolymer formulation (also known as a resin) that consists of a monomer or macromer with functional groups that can be polymerized or crosslinked, a photoinitiator that starts the polymerization process, a crosslinker that ensures sufficient crosslinking and mechanical stability of the 3D-printed part, a radical quencher, and a UV-absorber (e.g., a photosensitizer) to control the expansion of polymerization in the *Z*- and lateral directions. The choice of these resin components determines the material properties of the 3D-printed part, but also its functionality. For example, transparency, minimal feature size/resolution in the micrometer range, solvent resistance, and tailorable surface wettability are critical requirements for a material to be suitable for the PµSL printing of microfluidic devices. In general, with the introduction of homemade photopolymer formulations, these properties can be directly addressed. However, custom-made resins in 3D printing are not only interesting for microfluidic device fabrication, but also for robotics [70], self-healing objects [71], and electrically conductive parts [72], or for designing pneumatic grippers [73]. Critical parameters for tailoring resin properties, such that they are ready-to-use in PµSL printing, and for individual applications in microfluidics, are discussed in greater detail in Section 3.

### 2.3. Multimaterial Micro-Stereolithography

AM holds great promise for the single-step processing of functional materials. However, its potential is diminished by the fact that, although PµSL is a versatile tool for implementing flow cells with unique microchannel networks and complex 3D geometries, most examples rely on the use of single materials, which leads to monolithic flow cells with uniform material and microchannel properties. Although uniform material properties across a flow cell may provide sufficient functionality, e.g., to realize single-phase or multiphase continuous flow, the range of applications of microfluidic devices extends beyond these applications. For instance, multiparametric cell culturing, the design of multicompartment materials, such as (double) emulsions, biomolecule separation [74], and system integration in sensing applications may require tailored surface properties of the flow cell, particularly with spatial control. Conventionally, tailoring these surface properties, also with spatial control, commonly requires post-processing of the microchannel surface, e.g., by applying functional coatings.

**Figure 2:**
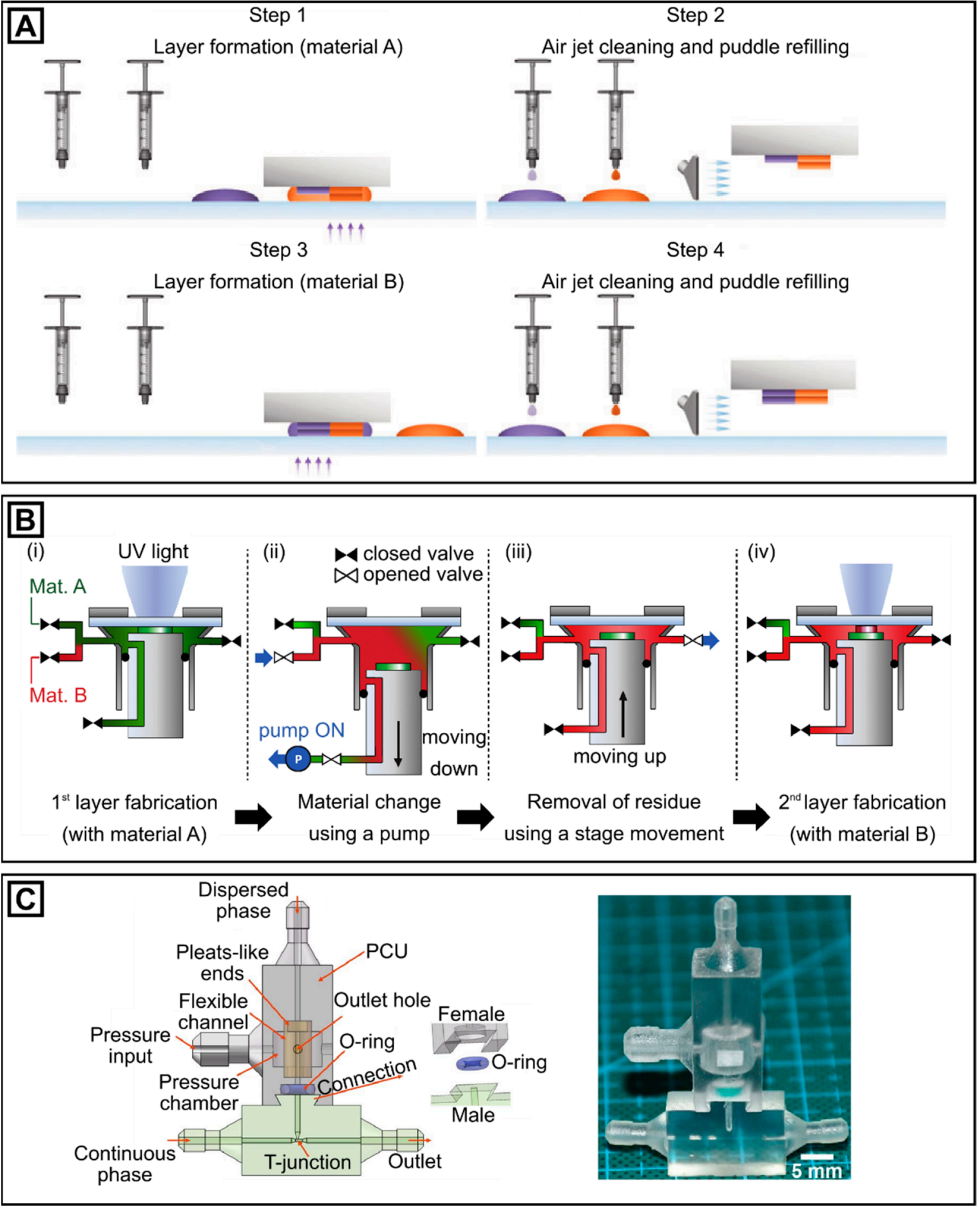
Multimaterial 3D printing based on PµSL. (**A**) Two different materials are processed together by a modified vat switching system. Syringes control the amount of resin placed on the glass. Pressurized air removes uncured resin to avoid introducing surface impurities when switching between resins. Adapted with permission from Ge et al. [79] (**B**) Resin exchange in multimaterial PµSL within a few seconds using dynamic fluid control of different resins within an integrated flow cell replacing the conventional resin vat. Adapted with permission from Han et al. [38] (**C**) Pre-fabricated microfluidic parts made from different materials assembled into a functional flow cell for emulsion formation. Adapted with permission from Ji et al. [80].

Thus, the design of advanced multifunctional microfluidic devices may require a shift toward processing multiple materials, ideally on a single PµSL platform. Although the implementation of multimaterial 3D printing has been realized in other AM techniques (e.g., DIW [75], FDM [76], and jetting [77]), only a few solutions for resin-vat-based polymerization techniques (e.g., multimaterial projection micro-stereolithography (MM PµSL)) are known. In general, three different approaches have been developed: (I) vat switching [78,79], (II) in situ material exchange through dynamic fluid control [38], and (III) the assembly of pre-printed parts (Figure 2) [80,81,82].

In 2011, Choi and coworkers introduced a custom-made 3D printer for processing different materials in a multimaterial approach [78]. They implemented a rotating-vat carousel system with four vats and successfully printed 3D structures from three different commercial resins. On the basis of this approach, Kowsari et al. constructed a novel digital light processing (DLP)-based micro-stereolithography approach, where different resins are placed on a glass plate, and the plate is moved to the printing position [79]. Different resins are processed, depending on the coordinates to which the plate moves (Figure 2A). The authors also shortened the time-consuming step of cleaning the printed layers and the glass plate by applying an air jet that removes the uncured resin within seconds before switching to the next resin; they claimed that their method using an air jet is approx. 56% faster than other approaches that use cleaning solutions.

In 2019, Han et al. proposed another solution for multimaterial PµSL printing [38]. In their system, a flow cell surrounding the printing platform is integrated with different microfluidic inflow ports—one for each resin. After a layer is printed with resin A, the flow cell is flushed with resin B to replace resin A within seconds. The purity of the printed layer with resin B was greater than 95%. This method was not only used for the 3D-printing of (nanoparticle-loaded) acrylate-based resins, but also for hydrogels. The researchers obtained multiresponsive objects with a layer thickness of 150 µm by combining different functional hydrogels being thermoresponsive and electroactive.

The third example of multimaterial processing of polymers that can be used in multifunctional microfluidic device design does not rely on MM PµSL, but it contributes to the idea of multifunctional 3D-printed devices and is, therefore, discussed herein. The idea of prefabricated discrete elements assembled after the printing process was first proposed by Malmstadt and coworkers in 2014 [81]. The authors designed a library of different microfluidic elements and connectors that were reversibly connected, with the objective of fabricating truly complex microchannel systems. With these microfluidic elements, the authors assembled flow-focusing devices and T-junctions for the production of emulsions with a channel cross-section of 750 µm. Following this approach, Duan et al. proposed a similar system that also incorporates different materials and takes different printing platforms into account [80]. As a major application, they used an elastic material for the pneumatic control of emulsion formation.

Although the aforementioned reports have contributed to the implementation of MM PµSL, high-resolution multimaterial 3D printing, in particular, remains an ongoing challenge.

### 2.4. Static Materials vs. Switchable and 4D Materials

PµSL printing is based on the idea of deconstructing a 3D computer-aided design (CAD) into 2D layers that are then added onto one another to yield the desired 3D object [83]. Although AM has evolved regarding accuracy, multiple material printing capabilities, and high printing speeds, conventional products, such as microfluidic devices, behave statically. Thus, although materials may be highly functional because of a complex microchannel architecture or a likewise complex surface functionalization, conventional materials in AM cannot respond to a change in environmental conditions over time. In so-called four-dimensional (4D) material printing, this time axis is considered in material functionality to obtain products with truly programmable properties, rather than static performance. Such a 3D-printed material can continuously adapt over its lifecycle, e.g., to mechanical changes and temperature, thus circumventing the need to redesign materials for individual sets of environmental conditions [84,85]. 4D materials can detect environmental stimuli and generate an advantageous response to the stimulus by changing either their material properties or their geometries, paving the way toward smart materials [86]. The external stimulus can be exerted physically, chemically, or biochemically. The response can be uniform regarding degradation, shrinkage, swelling, or a color change [87,88]. In addition, 4D-printed materials can be classified according to the type of external stimulus as thermo-, moisture-, photo-, electro-, magneto-, or pH-responsive. Applications that involve 3D-printed 4D materials include smart valves [89], microgrippers [90], drug delivery systems [91], energy-harvesting and storage systems [92], and functional organs [85,93].

Relevant examples in the field of 4D-printed biosciences include photoinitiated drug delivery [94], thermoresponsive surfaces for tissue printing [95], and magnetically actuated inchworm-inspired, biomimetic robots [96]. Along these lines, 4D printing has also influenced biomaterial design, where 4D materials have been used to more closely reflect the dynamic nature of living tissues (e.g., in the design of smart stents and implants) and organ printing (e.g., kidney, heart, and liver elements) [86,97,98,99].

Although 4D printing has been a revolution in the manufacture of dynamic structures, it commonly relies on conventional polymer materials that are known for their responsiveness and adaptiveness to enable the 3D-printing of 4D materials. One of the simplest examples is a material’s response toward humidity and salt concentration, as observed in, for example, 3D-printed hydrogels [97,98,99,100]. Another example is the plasmonic heating of 3D-printed polymer materials that convert light energy into heat, and then undergo a temperature-induced phase transition [99,101]. Furthermore, pH-responsive materials can swell or shrink in response to pH changes in the surrounding environment [102]. Among these materials, poly(*N*-isopropyl acrylamide) (PNIPAAm) is a prime example [103,104].

Quanjin et al. carried out a SWOT analysis of 3D and 4D printing technologies, evaluating their strengths, weaknesses, opportunities, and threats [105]. Their analysis indicated that the 3D printing of adaptive-responsive 4D polymer materials has the potential to soon provide highly engineered, intelligent materials that exhibit desirable changes in size, shape, and porosity, among other properties, because of the applied or recognized/identified triggers for future programmable materials that are fabricated by AM.

## 3. Requirements for PµSL Processability

The different printing techniques based on PµSL, homemade 3D printers and resins, along with the progress toward innovations in multimaterial printing, have already been discussed. Before focusing on applications, we will detail the polymerization techniques in PµSL and the resins themselves.

First, we need to distinguish between mass polymerization and solution polymerization. In the first case, the monomer itself is liquid such that this process does not require a solvent; in the latter case, the material is dissolved in a solvent before being used for 3D printing. In applications where solution polymerization is not based on an aqueous reaction mixture (e.g., 3D-printing of biocompatible hydrogels), but requires cytotoxic solvents that demand efficient post-processing to remove any solvent residues, bulk polymerization may be advantageous. Additionally, because of potential chain-transfer reactions with the solvent, the 3D-printed parts may be mechanically inferior as a consequence of incomplete crosslinking [106,107,108,109]. This review focuses on the vast field of mass polymerization of resins, although examples exist where solution polymerization has been used. For example, Wilking and coworkers proposed the 3D printing of microfluidic hydrogels containing spiral channels with submillimeter-scale cross-sections, where the spiral channels can be used to mimic the complex vasculature of living organisms [110].

### 3.1. Polymerization Techniques in PµSL

**Figure 3:**
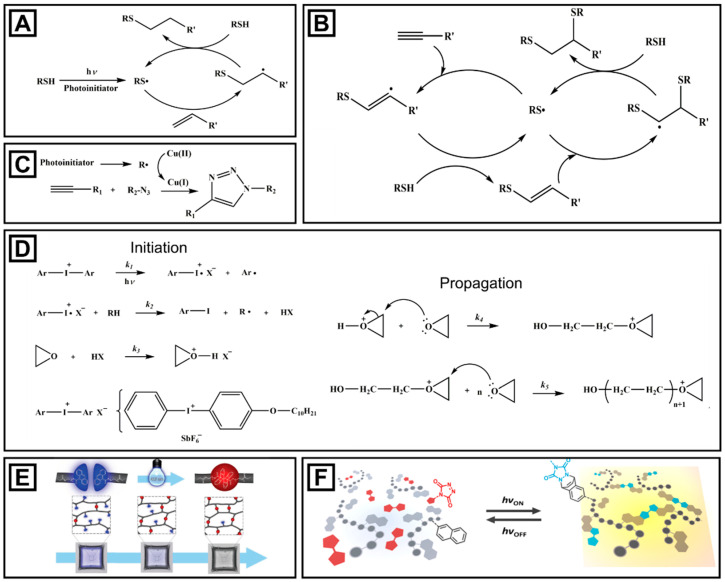
Potential crosslinking mechanisms in 3D printing: (**A**) thiol–ene (adapted with permission from Konuray et al. [123]), (**B**) thiol–yne (adapted with permission from Konuray et al. [123]), (**C**) copper-catalyzed azide–alkyne reaction (adapted with permission from Konuray et al. [123]), (**D**) cationic photopolymerization of epoxy monomers (adapted with permission from Crivello et al. [124]), and (**E**) cycloaddition dimerization of anthracene moieties (adapted with permission from Gernhard et al. [125]). (**F**) Schematic of covalent crosslinking of naphthalene containing PMMA polymers with bifunctional triazolinedione cross-linker in PµSL by visible-light-driven cycloaddition (adapted with permission from Houck et al. [126]).

SL is based on the photopolymerization of photosensitive monomers or macromers in the presence of a photoinitiator or a photoinitiator system that translates photolytic energy into reactive species, such as a radical or cation, triggering chain growth of the monomer or macromer into a polymer network and 3D-printed object, respectively [111,112,113,114,115]. Acrylate and methacrylate monomers or oligomers, as well as vinyl compounds, are commonly processed in SL-based printing via a radical chain-growth polymerization mechanism with initiation, propagation, and termination steps [111,113,115,116]. In addition, alkyl thiols can react in thiol–ene [116,117,118] (Figure 3A) or thiol–yne [119] (Figure 3B) addition reactions, which have been used for the 3D printing of soft robotics and self-healing materials [120]. Moreover, highly crosslinked networks are also accessible using multifunctional alkynes instead of vinyl monomers via a secondary thiol addition reaction with excess thiol groups [113,115]. Such 3D-printed structures could be degraded and erased under mild conditions with a well-defined trigger, such as ethanolamine, as Zieger et al. demonstrated using DLW [121]. The same research group also demonstrated that thioaldehydes can form network structures with thiol linkers, leading to disulfide-bridged polymer networks that can again be degraded via a thiol–disulfide exchange reaction with dithiothreitol [122].

Whereas free-radical polymerization conventionally provides chemically inactive polymer structures, the use of a living polymerization mechanism in PµSL enables the activation or deactivation of further polymerization of a 3D-printed object, which, in turn, enables the on-demand addition of features to an existing object. Jin and coworkers introduced both a photocontrolled reversible addition fragmentation chain transfer (photo-RAFT) polymerization [127] and a photoelectron/energy transfer reversible addition-fragmentation chain-transfer (PET-RAFT) polymerization [128] in DLW printing technology.

Epoxides and oxetanes can be cured through cationic photopolymerization, where thermally stable aryl iodonium and sulfonium salts act as cationic photoinitiators, which generate a mixture of cations, radical cations, and radical intermediates under UV irradiation [113,129,130]. Lantean et al. combined cationic polymerization with radical mechanisms through a hybrid monomer that contains both acrylic and epoxy functionalities to demonstrate the reactivity of the species under irradiation and evaluated the mechanical properties of the resultant prints [131]. Additionally, Zhao et al. introduced dual photopolymerization systems, such as acrylate silicone–epoxy hybrid resins in stereolithography printing, to achieve photopolymerization using a combination of free-radical and cationic polymerization mechanisms [132].

Light-induced step-growth polymerization methods do not necessarily require an initiator; they can proceed via a reaction of di- or trifunctional monomers, e.g., in photoinitiated azide–alkyne cycloaddition reactions (Figure 3C), as studied by Bowman and coworkers [133]. However, although this method can be coupled with spatiotemporal control over polymer material formation [133], its use in additive manufacturing has not yet been investigated in detail. Nonetheless, photoinitiated azide–alkyne chemistry has been used for post-processing surfaces of 3D-printed objects [134].

Diels–Alder (DA) reactions describe reversible cycloadditions between a conjugated diene and a dienophile under mild conditions (even room temperature [135]); however, at elevated temperatures, the retro-DA reaction occurs, which causes cleavage of the cyclic adduct (e.g., 110–140 °C) [136]. Li et al. reported PµSL printing of shape-memory and recyclable polyurethanes based on DA reactions [136]. They first prepared polyurethane acrylate with a DA adduct and then prepared photopolymer formulations with reactive diluents and photoinitiators to obtain a series of resins.

In another example of a photo-DA reaction, triazolinediones (TADs) were reacted with naphthalenes, yielding an unprecedented dynamic polymer system that could reversibly switch from a covalently crosslinked material in the presence of light to a viscoelastic liquid (Figure 3F) [126]. Houck et al. implemented a backbone functionalization of poly(ethylene glycol) with TADs, which could form polymer networks under UV irradiation in the absence of a photoinitiator. Moreover, the 3D-printed structure could be readily erased by water without requiring a change to acidic or basic reaction conditions [137]. In another set of cycloadditions, anthracene moieties were linked in a photodimerization via [4+4] cycloaddition under irradiation (λ > 300 nm) to yield DLW-printed polymer objects in the dark (Figure 3E) [125]. Finally, Khademhosseini and coworkers recently demonstrated the careful selection of crosslinking methods to balance the mechanical and chemical properties, and the response of living cells to 3D-printed polymer hydrogels [138].

The combination of UV light, radicals, and potentially uncured, cytotoxic resin components in 3D-printed polymer materials results in a challenging environment for advanced applications in cell biology and cell-free biotechnology because stereolithography techniques generally rely on a UV-light-induced crosslinking mechanism of photopolymer formulations. Thus, the use of resins and processing conditions that are cytocompatible is an ongoing challenge in PµSL printing.

### 3.2. Processing of Resins

**Scheme 2:**
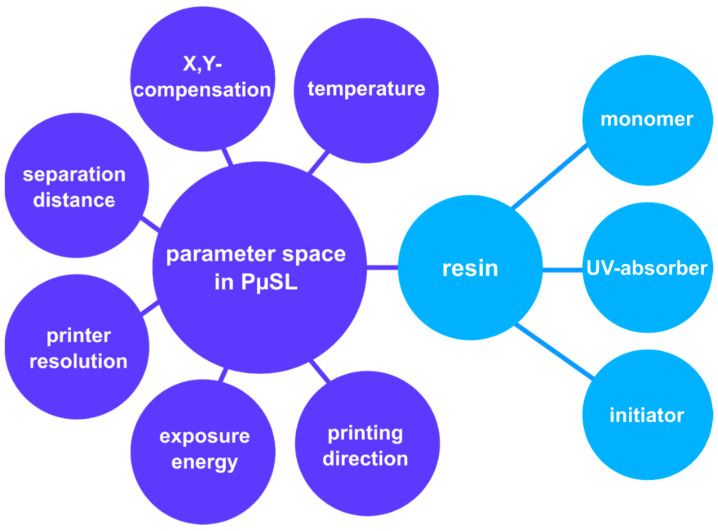
Potential parameter space in PµSL printing of, e.g., microfluidic devices.

A common feature of the aforementioned polymerization mechanisms is that they are suitable for use in PµSL. However, the radical polymerization of (meth)acrylates is mainly used with microchannel resolution down to 20 µm. However, achieving full control over the actual printing process of homemade resins requires not only knowledge about the polymerization mechanism, but also about the process parameters of the 3D printer (e.g., light intensity and exposure time) as well as the physicochemical properties and composition of the resin (e.g., concentration of components and additives, overall viscosity, and oxygen concentration). A good understanding of this complex parameter space is the basis by which the penetration depth of light into a photopolymer formulation—and, thus, the minimum feature size of a PµSL-printed object, such as a microfluidic device—can be controlled. Scheme 2 shows an overview of the most important parameters, which are, which are discussed in the following paragraphs.

The parameter space in PµSL is manifold; however, some of the parameters are considered to have more impact on the printing resolution than others. Here, we focus on the parameters that potentially increase the resolution of the 3D-printed structures the most, being set by the 3D-printing platform (intrinsic parameter) and the resin (extrinsic parameter). In detail, the exposure energy (I), separation distance (II), printer resolution (III), *X*,*Y*-compensation (IV), temperature (V), and the printing direction (VI) are considered to be intrinsic parameters that can be adjusted well before the printing process.

(I) The exposure energy is the result of the multiplication of the light intensity of the printer and the illumination in the printing process. It not only initiates the polymerization process itself, but also influences the polymerization depth of the material and, therefore, the layer thickness. For instance, with increasing the exposure energy, the polymerization depth increases and vice versa [139].

(II) The separation distance can be commonly adjusted in the 3D printer software. This parameter can also affect the dwell time of the last printed layer in atmospheric oxygen, as reported by our group in 2019 [20]. Oxygen inhibits the propagation of radical-driven polymerization, and it forms stable peroxy radicals that do not participate in the printing process and are considered to be dead-ends in the polymerization reaction [45].

(III) The lateral and vertical resolution of the printer strongly influences the printing resolution. The lateral resolution is the resolution in the *X*,*Y*-direction created by the optical setup—mainly the DMD and its pixel pitch—as well as by the use of optical (focusing) lenses [140]. By contrast, the vertical resolution in the *Z*-direction is set by the mechanical setup of the printer, and it also determines the possible layer thickness in the printing process. For example, the gap between the building platform and the transparent bottom of the vat is set to 20 µm. Therefore, the layer thickness is 20 µm, even if the polymerization depth of the resin is substantially greater, which then results in better adhesion of the before-printed layers. Nordin et al. proposed a custom-made PµSL printer with a lateral resolution of 7.6 µm, which they used for 3D-printing serpentine microchannels with a cross-section of 18 µm × 20 µm and a length of 3 mm [68]. Notably, the lateral resolution of the printer could not be translated into the highest resolution in a 3D-printed object because of potential overcuring and nonuniform light distribution; however, an in-depth discussion of these effects is beyond the scope of the current review [141].

(IV) The voxel compensation in the *X*,*Y*-plane is a parameter that can be specifically defined in PµSL printers. It describes the adjustment of the grayscales of the DMD to 3D-print microstructures that, otherwise, would lead to clogging, because the designs are not consistent with the printer’s lateral resolution. For example, a 25 μm cross-section of a microchannel would be either 20 µm or 30 µm in a cross-section fabricated with a printer that provides a lateral resolution of 10 µm. To overcome this mismatch, a printer’s grayscale can be varied from 0 (no illumination) to 127 (50% light intensity) to 255 (100% light intensity, full illumination). Here, the grayscales are adjusted between 0 and 255 to provide variations in light intensity for achieving partly polymerized voxels [20].

(V) An appropriate temperature during the printing process is critical to ensure the processability of a resin. Although most low-molecular-weight resins are liquids at room temperature, some are difficult to process because of the high viscosity of the starting materials. Increasing the temperature of the printer’s resin vat lowers the viscosity of the resin, which extends the range of materials that can be used in PµSL printing [72]. For instance, to process shape-memory polymers as a viscous melt (approx. 30 Pa s), Magdassi et al. heated the PµSL printer’s vat to 90 °C before 3D printing objects [142]. (VI) The sixth parameter to consider in PµSL printing is the orientation of the object (e.g., microfluidic device) at the printer platform. Structures within a polymer object can be orientated either along the *Z*-axis or along the *X*,*Y*-plane. An orientation along the *Z*-axis was shown to be favorable for achieving a higher resolution in micron-scale 3D printing, depending on the printer’s resolution and the layer thickness [20,143].

Beyond these intrinsic parameters, the extrinsic parameters of the to-be-processed material itself should also be considered. A resin typically comprises monomer(s)/oligomer(s), an initiator, a UV-absorber, a radical quencher, and a crosslinker, each of which contributes to the resin’s overall printability. In addition, the processability is highly dependent on the uniformity of the light intensity of the 3D printer. Lipson and Kurman showed that it is related to the curing depth $C_{d}$, which is expressed by Jacob’s working curve:

(1) $$C_{d}=D_{p}\ln\frac{E_{0}}{E_{c}}$$

where $D_{p}$ is the polymerization depth and $E_{0}$ and $E_{c}$ are the irradiation intensity and critical exposure to initiate the polymerization process, respectively [144]. Relying on this theoretical consideration, Ge and coworkers showed that the maximum light intensity is found at the center of a pixel and, therefore, results in a conical shape of the voxel [141]. Nordin et al. developed a mathematical model for the polymerization depth $z_{p}$ for a given exposure time $t_{p}$:

(2) $$z_{p}=h_{\propto }\ln\frac{t_{p}}{T_{c}}$$

Here, $h_{a}=\frac{1}{\alpha }$, where α′ is the resin’s absorption coefficient, which is unique for the chosen material, and it can be adjusted by the UV-absorber [145]. Zheng et al. and Gong et al. [45,68] showed that a UV-absorber reduces the reaction rate of the polymerization reaction by limiting the number of available photons, which resulted in a reduced polymerization depth and, thereby, enabled the layer thickness to be controlled and the resolution to be optimized [45,68]. Notably, the optical absorption spectrum of the UV-absorber must overlap with the optical emission spectrum of the light source to provide a sufficient reduction of the reaction rate. Interestingly, it has been shown, for a resin consisting of 1,6-hexanediol diacrylate, Sudan 1, and diphenyl(2,4,6-trimethylbenzoyl)phosphine oxide, that the concentration of the photoinitiator had limited effect on the polymerization process; e.g., it did not change the polymerization depth at all at photoinitiator concentrations greater than 2% [45]. In addition, increasing the light intensity and exposure time lead to greater layer thicknesses, and to an increase in the polymerization depth, respectively.

A potential issue that is associated to the post-processing of 3D-printed materials is volume shrinkage and, thus, changes in the object’s dimensions. While significant shrinkage occurs during the processing of, for example, ceramics, this challenge is almost negligible in PµSL of polymer formulations. For instance, Wenjuan and coworkers showed, in 2017, that the shrinkage of their prints was less than 2% [146]. In another example, a commercial material supplier claims a shrinkage smaller than 0.5% [147].

## 4. Applications of PµSL in Microfluidics

The fabrication of microfluidic devices that are based on PµSL is discussed in greater detail later in this section; first, we briefly highlight the potential of PµSL for fabricating microstructured polymer materials with a complex 3D geometry for other applications. Because 3D-printed materials in PµSL often exhibit substantial toxicity due to additives, such as the UV-absorber Sudan I, they are not suitable for most biomedical applications. To address this problem, Männel et al. [148] and Warr et al. [149] developed resins that were based on poly(ethylene glycol) diacrylate (PEGDA) exhibiting sufficient cell viability and proliferation in long-term cell culture experiments. Männel et al. [148] proposed a combination of PEGDA, poly(ethylene glycol) methyl ethyl methacrylate, Sudan 1, and diphenyl-(2,4,6-trimethylbenzoyl)phosphine oxide to cultivate human umbilical vein endothelial cells for 24 days. Washing the 3D-printed object after the printing process in phosphate-buffered saline removed Sudan 1, dramatically reducing the cytotoxicity of the 3D-printed cell culturing substrates. Warr et al. [149] used a similar resin based on PEGDA and avobenzene as the UV-absorber. They washed their 3D-printed object in ethanol for 12 h to reduce the cytotoxicity of the material and then subjected the object to a plasma treatment to improve cell adhesion. On the other hand, Kotz et al. used a commercial SL printer for processing a photocurable silica nanocomposite to fabricate transparent fused glass devices with a resolution of approximately a few tens of micrometers. The 3D-printed parts were transformed into glass components through a stepwise heat treatment, which resulted in a smooth surface with a roughness of only a few nanometers [150].

Another field of application of PµSL toward the fabrication of complex 3D microstructures is soft robotics. For example, Boydston and coworkers built three-armed pneumatic grippers from a homemade elastomer with a minimal tensile stress of 0.104 MPa [73]. Magdassi and coworkers formulated a homemade resin that was based on aliphatic urethane diacrylates to fabricate highly stretchable 3D-printed objects for soft robotics [72], and Peele et al. used a commercially available elastomer resin to fabricate soft actuators as a model for artificial muscles [151]. These examples highlight the broad range of applications that are accessible with PµSL. The following subsections focus on the application of PµSL in microfluidics, with an emphasis on 3D-printed microfluidic devices for forming single and double emulsions.

### 4.1. Microfluidics: Applications and Functional Components

#### 4.1.1. Applications in Microfluidics

Before focusing on microfluidic devices for forming emulsions, we note other applications of microfluidics and functional microfluidic modules already realized by PµSL. In 2018, Choi and coworkers 3D-printed a microviscometer using a stereolithography-based printer to measure blood viscosity [152]. In the same year, Zhang’s group developed a hybrid modular system for generating droplets in glass capillaries. Their system comprised three parts: (I) a top module for the dispersed phase, (II) a glass capillary for emulsion formation, and (III) a bottom module for the continuous phase; components (I) and (III) were 3D-printed by PµSL [153].

The fast, efficient mixing of multiple flows is another important subfield in microfluidics. Here, the advantage of 3D printing over conventional microfluidic device fabrication methods again lies in the ability to create microchannels in different layers in a single step [154,155,156,157]. Eijkel et al. 3D-printed a microfluidic device with five inflow ports that were filled with collagen to monitor the concentration profiles of solutes to mimic targeted drug delivery [158].

Controlling the merging of microdroplets is critical for mixing small, individual volumes in chemical and biological analyses. To this end, Thoroddsen and coworkers 3D-printed a microfluidic device to merge droplets passively via pillar blocks inside microchannels [159]. Wagner’s group proposed a 3D-printed sensor system for monitoring cell growth inside microfluidic devices PµSL-printed using the commercial resin PlasCLEAR™ [160]. Before the actual cell-culturing experiment, the 3D-printed device was immersed in the cell culture medium for two days [160]. Similarly, Folch and coworkers developed a transparent resin based on low-molecular-weight PEGDA for the long-term culturing of sensitive neuron cells [161]. Jeon et al. used a 3D-printed cylindrical microchannel to develop an immunomagnetic flow assay for the detection of pathogenic bacteria with high sensitivity and high capacity [162,163]. In another example, Alessandri et al. introduced a microfluidic device that generates submillimeter hollow hydrogel spheres encapsulating human neural stem cells [164].

#### 4.1.2. Functional Components

Focusing on functional elements in microfluidics to control fluid flow inside microchannels, such as valves and pumps, Au et al. used the commercial resin WaterShed XC 11122™ to transfer Quake’s valve design [165] into 3D-printed flow cells that are suitable for cell culture [166]. The authors chose WaterShed™ because of its ability to provide transparent flow cells that do not swell in water and that meet the minimum standards for biocompatibility. However, like materials 3D-printed with other commercial resins, those 3D-printed with WaterShed™ lack resolution, which results in microfluidic channels in the range of millifluidics rather than microfluidics. Lee et al. used a homemade resin based on PEGDA to 3D-print Quake’s valves to overcome the issue of limited resolution in PµSL [167]. Membranes with thicknesses of 25 µm and even 10 µm were successfully fabricated, and the valves were closed pneumatically with pressures between approx. 20 kPa and 40 kPa. In addition, arrays of 64 valves were 3D-printed in an 8-by-8 fashion to demonstrate the scalability and reliability of the fabrication approach. However, because the Young’s modulus of the material was approx. 130 MPa as compared to >1 MPa for PDMS [168], the authors adapted their flow-cell design to ensure sufficient sealing. Because of this poor material flexibility compared with that of PDMS, Männel et al. proposed a homemade resin based on tri(propylene glycol) diacrylate, which resulted in a reduction of the Young’s modulus to approx. 15 MPa [169]. In addition, microchannels that were 3D-printed from this homemade resin exhibited cross-sections as small as 40 µm, which is similar to the minimum feature size and resolution achieved in PDMS-based microfluidic devices. The authors demonstrated the applicability of their resin formulations for microfabricating two different valve designs: Quake’s valves (cf. above) and a design that is based on the work of Nordin and coworkers [170]. In that original work from 2015, the authors 3D-printed valves via PµSL as a proof of concept [139]. The microchannels had a cross-section of 250 μm × 350 µm with a single-layer valve that was closed by applying approx. 74 kPa to deflect the membrane, which was successfully demonstrated for 800 actuations. By adding a second thermally actuated initiator to their resin formulations, Nordin and coworkers further improved the durability of the valves for as many as 1,000,000 actuations [171]. Moreover, with five-pump actuation, they could use their membranes as a microfluidic pump with flow rates as high as 40 µL min^−1^.

The aforementioned examples demonstrate that researchers have already successfully translated the design of microfluidic modules from conventional fabrication techniques into PµSL. These modules and their material properties are critical for a wide range of microfluidic applications and they represent a decisive step for the future development of 3D-printed complex microfluidic devices.

### 4.2. Single Emulsion and Double Emulsion Formation in PµSL-Printed Devices

#### 4.2.1. Single Emulsion Formation

Microfluidic devices can be operated in two fundamentally different modes: two fluids form a continuous co-flow or, in the case of distinct interfacial tension between two fluids, these fluids form a segmented flow and droplets, respectively [172]. The physics behind droplet formation is beyond the scope of this review; interested readers are referred to several excellent related publications [172,173,174].

Normally, all of the microchannels inside a microfluidic device are within the same layer when a conventional, single-layered master structure, also referred to as a planar microchannel geometry, is used. In these microfluidic devices, surface wettability may be critical to the formation of a certain type of emulsion (i.e., water-in-oil or oil-in-water). When PµSL printing is used as the fabrication method of choice, complex 3D structures in which surface wettability becomes negligible can be achieved because a nonplanar microchannel geometry can be realized [175].

**Figure 4:**
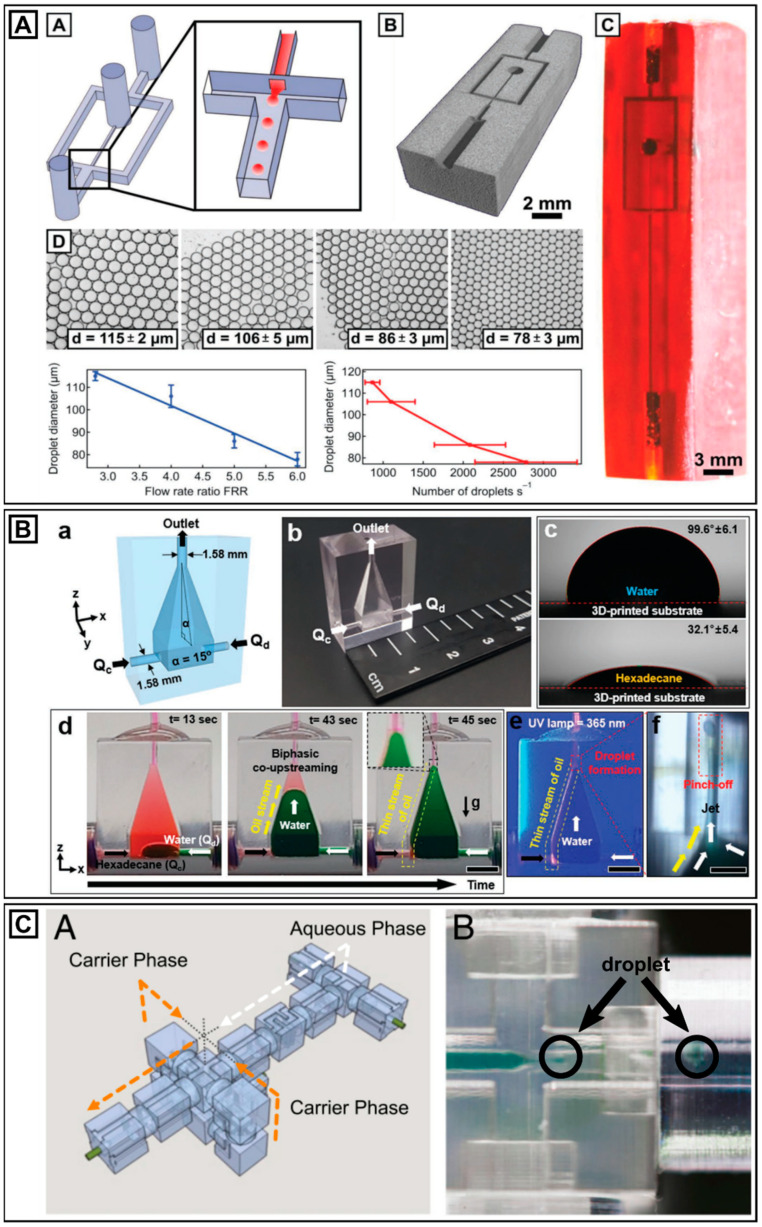
Microfluidic devices for emulsion formation, fabricated via PµSL. (**A**) Nonplanar flow-focusing device for forming both oil-in-water and water-in-oil emulsions. Droplet size is manipulated by adjusting the flow rate ratio (FRR). Adapted with permission from Thiele et al. [20] (**B**) 3D-printed chimney-shaped millifluidic device for producing emulsions with diameters as small as 60 µm. Adapted with permission from Kim et al. [177] (**C**) Interconnected discrete 3D-printed moduli, resulting in a flow-focusing device for microdroplet formation. Adapted with permission from Malmstadt et al. [81].

Following the idea of nonplanar devices for emulsion formation without tailored microchannel wettability, Thiele’s group used the commercial resin R11™ and a commercial PµSL printer to fabricate monolithic microfluidic devices for droplet formation (Figure 4A) [20]. Specifically, flow cells with a microchannel cross section of 75 µm for the dispersed phase and 200 μm × 300 µm in the cross-section for the continuous phase were fabricated. The devices were used for the formation of both water-in-oil and oil-in-water emulsions inside the same devices, which is a substantial advantage of nonplanar complex 3D structures over conventionally fabricated microfluidic devices. The droplets then served as templates for forming microparticles with diameters as small as 93 µm via UV-induced polymerization of a prepolymer solution inside the droplets. Zhang et al. proposed a similar approach in 2016. Here, the microchannel dimensions were larger: 600 µm in diameter for the dispersed phase and 1000 µm for the continuous phase [176]. While these examples are based on 3D-printed nonplanar geometries, Kim and coworkers demonstrated a planar droplet maker [43]. They showed that the diameter of the circular channel was 400 µm and that water-in-oil emulsions could be formed at rates between 20 and 80 droplets per minute, depending on the flow rate ratio. Finally, they 3D-printed a multilayered microfluidic device with two flow-focusing junctions for parallel droplet formation. The inflow port of the dispersed phase was located in a different layer from the inflow port of the continuous phase, highlighting the great flexibility in 3D design.

Hwang et al. PµSL-printed a millifluidic device with a chimney-shaped geometry consisting of a cubic bottom part and a pyramidal upper part (Figure 4B) [177]. The fluids were introduced from opposite directions for parallelization of droplet formation. Interestingly, although all of the microchannels were designed to be 1.58 mm in diameter, which is far from typical dimensions of microfluidic channels at first glance, depending on the flow rate ratio and the apex angle α, the droplet sizes could be varied from 36 µm to 446 µm [178]. The authors exploited the ease of fabrication in PµSL printing to fabricate four parallelized chimney-like microchannels for simultaneous droplet formation. Their approach has potential for widespread industrial use because of the excellent material uniformity and the high flow rates (as high as 70 mL min^−1^) with no device leakage. In 2015, Femmer et al. reported the preparation of parallelized microfluidic droplet makers with flow-focusing geometry for the high-throughput production of emulsions via PµSL [179]. Their device comprised 28 parallelized droplet makers and produced 500 µm-diameter emulsion droplets at a rate of 3 L h^−1^; the droplets were transformed into microgels by a photochemical crosslinking reaction.

The common limitation of the aforementioned examples is that the microfluidic modules are fabricated as monoliths. The flow cell design needs to be adapted to implement additional microfluidic modules (e.g., a second consecutive junction or a valve), which usually requires additional printing steps. To address this problem, Malmstadt and coworkers developed a library of standardized components and connectors to assemble truly complex microfluidic systems in three dimensions (Figure 4C) [81]. The authors stated that their approach is superior to traditional methods with respect to cost and maintenance. The discrete elements are reversibly connected in a manner that is similar to LEGO™ bricks. To benchmark their concept, the authors first fabricated a T-junction device for forming droplets with a microchannel cross-section of 750 µm, where the connectors between fluidic elements were 1000 µm. By implementing four-way liquid processing, they also parallelized droplet formation, which indicated that their approach is not limited to 2D module assemblies. Eventually, by replacing the T-junction element with a cross-junction module, they assembled a flow-focusing device operating with a continuous-phase flow rate of 5 mL h^−1^ for forming water-in-oil emulsions.

Seo et al. [180] reported another example of a microfluidic device. They developed a drop maker that consists of two main components: an internal thread (nut) with a vertical T-junction and an external thread (screw). When rotated, the screw moved upward or downward, adjusting a gap at the T-junction, which enabled the droplet size to be varied from 39 µm to over 1000 µm within the same flow cell. Similar to the device proposed by Hwang et al. [177], that of Seo et al. [180] enabled the size of the droplets to be adjusted by more than two orders of magnitude without changing the CAD of the device.

#### 4.2.2. Double Emulsion Formation

**Figure 5:**
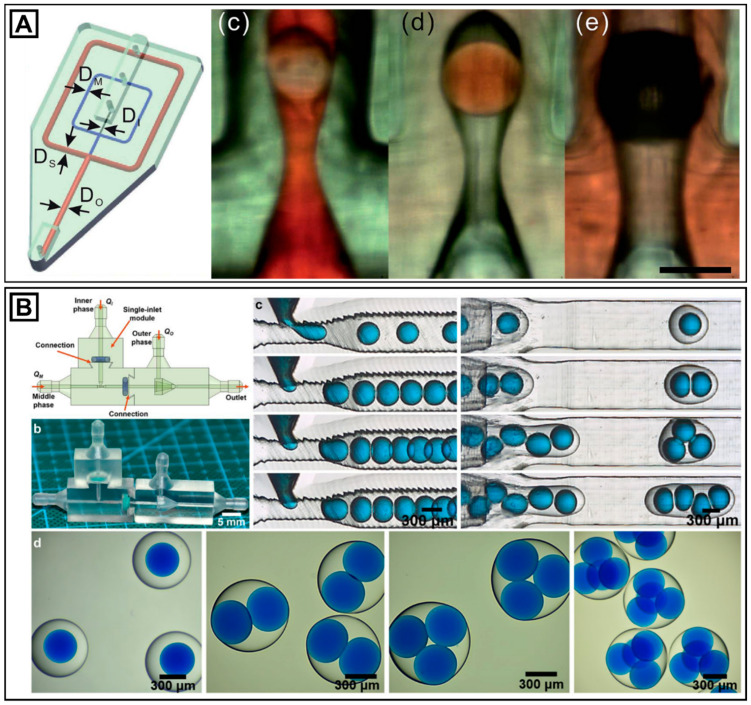
3D-printed microfluidic devices for forming double emulsions. (**A**) Nonplanar microfluidic device design for producing oil-in-water-in-oil, water-in-oil-in-water, or air-in-oil-in-water emulsions. Adapted with permission from Thoroddsen et al. [176] (**B**) Device for forming double emulsions, assembled from 3D-printed flow cell modules. The number of encapsulated liquid cores could be tuned by adjusting the flow rates. Adapted with permission from Duan et al. [80].

In recent years, not only simple single emulsions, but also microfluidically prepared double emulsions, which are droplets inside droplets, have attracted widespread interest, because the number of liquid cores as well as the core and shell volume can be well controlled [6]. Double emulsions have been used as templates for capsule and vesicle formation and they play a key role in drug delivery [3,181,182], food applications [4,183], and separation processes [184]. The production of microfluidic devices for forming double emulsions has been challenging because of the need for spatially controlled microchannel surface wettability. Although several approaches for overcoming the necessity of microchannel wettability patterning have been reported, they require extensive experience on the part of the user [185,186,187]. Here, 3D printing offers a facile route to circumvent this challenge, and eventually yields easy-to-fabricate devices for double emulsion formation. As described, changing the channel structure from a planar to a nonplanar 3D architecture results in negligible surface wettability, because the inner phase for droplet formation is fully surrounded by the outer phase. Zhang et al. reported a 3D-printed nonplanar, monolithic device with two consecutive flow-focusing junctions for forming both water-in-oil-in-water and oil-in-water-in-oil double emulsions (Figure 5A) [176]. Their device contained circular channels with diameters ranging from 600 µm (inner phase) to 1000 µm (middle phase) and 2000 µm (outer phase). By forming both types of double emulsions in the same device, they demonstrated the convenience of 3D printing as compared to conventional fabrication methods, where different microfluidic devices are often used for different types of emulsions. Inspired by this approach, Männel et al. developed monolithic double emulsion devices with improved microchannel resolution. Their flow cells contained rectangular microchannels with cross sections of 100 µm (inner phase) and 300 µm (middle and outer phases) [20]. They also demonstrated that both types of double emulsions could be generated, and tuned the flow rates to encapsulate either one or two smaller droplets in the outer double emulsion droplet.

In 2018, Takeuchi’s group published the assembly of a coaxial microfluidic device for forming single and double emulsions [188]. Their modules were assembled via screw threads, and they could be easily rinsed after use. By assembling an axisymmetric flow-focusing device, they generated water-in-oil-in-water double emulsions with a diameter of 370 µm for the outer droplet and 250 µm for the inner droplet at flow rates of 10 µL min^−1^ (inner phase), 50 µL min^−1^ (middle phase), and 500 µL min^−1^ (outer phase). In addition, by decreasing the outer phase’s flow rate to 200 µL min^−1^, multiple inner water droplets with an average diameter of 234 µm were encapsulated in the outer droplet with an average diameter of 835 µm. Ji et al. proposed another modular microfluidic device using multimaterial 3D printing [80]. They used two different 3D printers—a PolyJet and a Form-2—for device fabrication. With the first printer, they 3D-printed an elastic pneumatic control unit; with the Form-2 printer, they 3D-printed microfluidic modules, such as T-junctions. The different parts were connected via a notch structure, and an O-ring between the modules prevented device leakage. By connecting the pneumatic control unit with the T-junction module and the co-flow structure module, the authors formulated water-in-oil-in-water double emulsions (Figure 5B). Before generating the actual double emulsions, the T-junction module’s surface was rendered hydrophobic and the surface of the co-flow channel was rendered hydrophilic. Finally, the authors demonstrated full control over the number of encapsulated inner droplets from one to four encapsulated compartments.

## 5. Conclusions

In this review, we presented an overview of the state of the art in microfluidic device fabrication via PµSL, focusing on droplet formation as the target application. Although the devices can be fabricated as monolithic or modular, each approach has advantages and disadvantages that must be considered. We hope that this overview helps researchers that are interested in this field in their decision-making process for identifying the most suitable approach for their studies. While we evaluate that PµSL printers and resins in the market have reached a state where they will be able to revolutionize the fabrication of microfluidic devices, they suffer from inherent limitations, like poor transparency or processability. Thus, custom-made solutions on both the process side and the materials side are desired for full control over essential printing and material parameters, such as the minimum feature size, transparency, elasticity, and biocompatibility. In addition, the applications of microfluidic devices are so diverse that multimaterial printing using PµSL is an important focus of research for achieving different material and surface properties within flow cells. Because multimaterial printing is still in its early stages, additional studies for improving the system design, resolution, and material range are needed.
